# Supplementary material for: The genome sequence of the Red-tipped Clearwing, Synanthedon formicaeformis (Esper, 1783)
Source: Wellcome Open Res. 2026 Feb 20;8:260. Originally published 2023 Jun 21. [Version 2] doi: 10.12688/wellcomeopenres.19487.2 (PMC13033129; doi:10.12688/wellcomeopenres.19487.2)
Supplement: Peer review history

# Reviewer report: Reviewer response for version 2

**DOI:** 10.21956/wellcomeopenres.28654.r150232

**Published:** 2026-03-28

**Authors:** Jonathan Badger

**Affiliations:** 1 National Cancer Institute, Misrata, Libya; 2 National Cancer Institute Center for Cancer Research (Ringgold ID: 272101), Bethesda, Maryland, USA

The authors have sequenced the genome of the red-tipped clearwing ( *Synanthedon formicaeformis), *a moth found in Europe and the Near East. The genome was sequenced from a single male representative from the UK and 31 sequence scaffolds representing the autosomes and the Z sex chromosome. There was a second haplotype detected but the assembly deposited is of a single haplotype. The assembly statistics and BUSCO analyses look in general good. The assembly has been deposited in INSDC. The annotation is not currently available but apparently will be in the future.

 My only complaint with the manuscript is one that has been brought up in the first round of reviews by others  -- that of the missing annotation. I'm actually a bit confused on this point because the manuscript says it will be available, but the author's note implies that it is already available. Which is it?

Are sufficient details of methods and materials provided to allow replication by others?

Yes

Is the rationale for creating the dataset(s) clearly described?

Yes

Are the datasets clearly presented in a useable and accessible format?

Yes

Are the protocols appropriate and is the work technically sound?

Yes

Reviewer Expertise:

I am a computational genomics researcher who has worked on genomes of bacteria, fungi, protists, and insects.

I confirm that I have read this submission and believe that I have an appropriate level of expertise to confirm that it is of an acceptable scientific standard.

# Reviewer report: Reviewer response for version 1

**DOI:** 10.21956/wellcomeopenres.21585.r132105

**Published:** 2025-09-24

**Authors:** Aqeel Alyousuf

**Affiliations:** 1 University of Basrah, Basrah, Iraq

The manuscript presents a high-quality genome assembly of *Synanthedon formicaeformis*, generated using a complementary sequencing strategy that combines PacBio HiFi reads, Illumina RNA-Seq, and Hi-C scaffolding. The introduction effectively situates the study within the species’ ecological and geographical context; the sampling procedures are described with clarity, ensuring both traceability and transparency.

 The genome assembly is robust and carefully curated, with high-quality metrics (321.2 Mb, N50 of 11.7 Mb, BUSCO completeness (version 5.3.2) of 97.8%). The authors provide thorough DNA and RNA quality control, including the mitochondrial genome, and clearly address ethical and legal considerations, reflecting the rigor and reliability of the work.

 A minor recommendation is to introduce the full species name at first mention and subsequently use the abbreviated form ( *S. formicaeformis*) in accordance with standard taxonomic practice.

Are sufficient details of methods and materials provided to allow replication by others?

Yes

Is the rationale for creating the dataset(s) clearly described?

Yes

Are the datasets clearly presented in a useable and accessible format?

Yes

Are the protocols appropriate and is the work technically sound?

Yes

Reviewer Expertise:

Entomology

I confirm that I have read this submission and believe that I have an appropriate level of expertise to confirm that it is of an acceptable scientific standard.

# Reviewer report: Reviewer response for version 1

**DOI:** 10.21956/wellcomeopenres.21585.r81084

**Published:** 2024-05-21

**Authors:** Diler Haji

**Affiliations:** 1 Department of Integrative Biology, University of California, Berkeley, USA

Did the authors conduct an independent assessment of the number of chromosomes in this species before genome sequencing? Was this done in a previous study that hasn’t been mentioned in the present paper? This would be useful to include.

 Under the “Genome Sequence Report '' section, the authors state that the mitochondrial genome was also assembled and identified among the assembly contigs. It would be helpful to include the total length and completeness of the mitochondrial genome in this section. 

 The authors state that “the mitochondrial genome was assembled using MitoHiFi, which runs MitoFinder or Mitos”. Please be clear about which program was used for assembling the mitogenome.

 The authors mention that they produced transcriptome data from abdominal tissue. However there is no analysis of transcriptome data included in the paper. The authors should include an analysis of transcriptome data, and ideally an annotation of their genome using these data. In addition, the authors should take into consideration the biases associated with using abdominal tissue, including the presence of gut microorganisms.

Are sufficient details of methods and materials provided to allow replication by others?

Yes

Is the rationale for creating the dataset(s) clearly described?

Yes

Are the datasets clearly presented in a useable and accessible format?

Partly

Are the protocols appropriate and is the work technically sound?

Yes

Reviewer Expertise:

Evolutionary genomics

I confirm that I have read this submission and believe that I have an appropriate level of expertise to confirm that it is of an acceptable scientific standard, however I have significant reservations, as outlined above.

# Response

**Published:** 2026-02-04

**Authors:** Tree of Life Team Sanger

**Affiliations:** Wellcome Trust Sanger Institute, UK

Thank you for reviewing this data note. Information on chromosome number is available for the genus and family, and was used as contextual background during assembly and curation. We did not perform an independent cytogenetic analysis for this study. We have now added the length and INSDC accession of the mitochondrial genome to the *Genome sequence report* section and to Table 1. We have clarified that the mitochondrial genome was assembled using **MitoHiFi** with **MitoFinder** for gene annotation, and have corrected the wording accordingly. RNA-Seq data were generated to support genome annotation and were not intended as a standalone transcriptomic study. The genome has been annotated by EMBL-EBI since version 1 of this data note, and links to the annotation resources are provided in the manuscript. An analysis of transcriptome expression patterns and tissue-specific biases is outside the scope of this data note.   Tree of Life team, Wellcome Sanger Institute
